# Supplementary material for: Correlates of Physical Activity of Children and Adolescents with Autism Spectrum Disorder in Low- and Middle-Income Countries: A Systematic Review of Cross-Sectional Studies
Source: Int J Environ Res Public Health. 2022 Dec 5;19(23):16301. doi: 10.3390/ijerph192316301 (PMC9738504; doi:10.3390/ijerph192316301)
Supplement: Supplementary file 1 [file ijerph-19-16301-s001.zip › ijerph-2031642-supplementary.pdf]

Table S1. Criteria for evaluating internal validity of reported studies using quantitative methodologies [48,49].

	Questions	Evaluation	
**Purpose**	Q1: Was the study purpose stated clearly?	1=Yes	0=No
Study background	Q2: Was the relevant background literature reviewed?	1=Yes	0=No
Study design	Q3: Was the design appropriate for the research question?	1=Yes	0=No
Sampling	Q4: Was the sample described in detail?	1=Yes	0=No
	Q5: Was the sample size justified?	1=Yes	0=No
	Q6: Was informed consent obtained? (if not described, assume No)	1=Yes	0=No If not applicable, assume 3
Measurement	Q7: Were the outcome measures reliable? (if not described, assume No)	1=Yes	0=No
	Q8: Were the outcome measures valid? (if not described, assume No)	1=Yes	0=No
	Q9: Was the method described in detail	1=Yes	0=No
Data analysis	Q10: Were results reported in terms of statistical significance?	1=Yes	0=No
	Q11: Were the analysis methods appropriate?	1=Yes	0=No
	Q12: Was importance for the practice reported?	1=Yes	0=No
	Q13: Were any drop-outs reported?	1=Yes	0=No If not applicable, assume 3
Conclusions	Q14: Were the conclusions appropriate given the study methods?	1=Yes	0=No
Implications	Q15: Are there any implications for practice given the results of the study?	1=Yes	0=No
Limitations	Q16: Were limitations of the study acknowledged and described by the authors?	1=Yes	0=No

Table S2. Results of study quality evaluation using the McMaster Critical Review Form for Quantitative Studies.

Study	Items									Score	
	Purpose	Study Background	Study Design	Sampling	Measurement	Data Analysis	Conclusions	Implications	Limitations	Raw	%
Pan & Frey (2005) [56]	Y	Y	Y	3Y	3Y	4Y	Y	Y	Y	16	100
Pan & Frey (2006) [57]	Y	Y	Y	3Y	3Y	4Y	Y	Y	Y	16	100
Pan (2008 a) [31]	Y	Y	Y	2Y	3Y	3Y	Y	Y	Y	15	93.8
Pan (2008 b) [41]	Y	Y	Y	2Y	3Y	4Y	Y	Y	Y	15	93.8
Pan (2009) [58]	Y	Y	Y	2Y	2Y	4Y	Y	Y	Y	14	87.5
Pan et al. (2011a) [42]	Y	Y	Y	2Y	2Y	4Y	Y	Y	Y	14	87.5
Pan et al. (2011b) [59]	Y	Y	Y	Y	Y	4Y	Y	Y	N	11	68.8
Pan et al. (2011c) [60]	Y	Y	Y	2Y	3Y	4Y	Y	Y	Y	15	93.8
Memari et al. (2013) [61]	Y	Y	Y	2Y	3Y	4Y	Y	Y	Y	15	93.8
Memari et al. (2015) [62]	Y	Y	Y	Y	2Y	4Y	Y	N	Y	12	75
Pan et al. (2015) [43]	Y	Y	Y	2Y	2Y	4Y	Y	N	Y	13	81.3
Bicer & Alsaffar (2016) [63]	Y	Y	Y	Y	Y	4Y	Y	Y	Y	12	75
Pan et al. (2016) [40]	Y	Y	Y	2Y	2Y	4Y	Y	Y	Y	15	93.8
Memari et al. (2017) [23]	Y	Y	Y	2Y	3Y	4Y	Y	Y	Y	15	93.8
Pan et al. (2021) [24]	Y	Y	Y	2Y	3Y	4Y	Y	Y	Y	15	93.8
